# ***Cultural psychology of education***: ***approaches and strategies***

**DOI:** 10.1007/s12124-022-09707-2

**Published:** 2022-06-23

**Authors:** Giuseppina Marsico, Maria Virginia Machado Dazzani

**Affiliations:** 1 Department of Human, Philosophichal and Educational Sciences, University of Salerno, via Giovanni Paolo II, 134, 84084 Fisciano, SA Italy; 2 Federal University of Bahia, Salvador, Brazil

**Keywords:** Culture, Education, Psychology, Educational Settings, Values.

## Abstract

Education is a human activity that is continuously developing. This is the core of the Cultural Psychology of Education framework. There are no ready-made, perpetual solutions that work everywhere and for every person. Two central themes dominate this chapter: (1) Education is cultural in nature, and (2) Educational Psychology is culturally guided. The Cultural Psychology approach to education reflects the interdisciplinary nature of Educational Psychology and informs its application in a variety of cultural contexts. Cultural Psychology of Education is international and global, promoting cultural sensitivity within the educational environment and the idea that in any society, the application of knowledge in the area of education is locally based. Nevertheless, the field strives to contribute to the discussion about education on a global scale. Cultural Psychology of Education has a necessary moral commitment to the dignity, integrity and diversity of human life. The promotion and protection of the well-being of individuals and diverse human communities is a core aspect inherent in both the theoretical applications and the professional practices.

## Introduction

Education is a human activity undergoing continuous development. This is the core of the Cultural Psychology of Education framework. There are no ready-made and perpetual solutions that work everywhere and for everyone. For instance, the World Economic Forum’s Global Challenge Initiative on Employment, Skills and Human Capital published its 2017 report on the Future of Jobs project, and according to this report, 65% of current students will have jobs that are not yet in existence today. A false assumption exists that certain cultural or geographical areas are more developed in teaching and learning practices and that such areas correspond to the wealthiest nations. Innovation can spring from anywhere and must be adequately cultivated, not only economically but also in terms of cultural grounding and collective efforts. From a Cultural Psychology perspective, the education of the future must be regarded as global in its vision but local in its solutions. An ongoing reform movement exists in education in locations such as China, Italy, Nordic countries and Brazil, representing attempts to reform school systems either at the primary or secondary education levels. There is also a significant debate about the relationship between the “school for all” principle and the neo-liberalist and job-market-oriented approaches to education. This debate has been highlighted in several of the major journals in the field of Education. However, education is an open system in constant development; accordingly, the question of “What’s next?” remains crucial. What is at stake today, and what may be even more relevant in the future, is the capability of formal educational systems to provide an effective environment. This applies not only to the acquisition of different kinds of skills or to securing the ongoing development of creativity and innovation. Crucially, a vision of a future-oriented education applies to the supported development of students’ full potential beyond social expectations. Two central themes dominate this chapter: (1) Education is cultural in its nature, and (2) Educational Psychology is culturally guided.

## Cultural psychology of Education curriculum

We can point to three aspects that characterise the contribution of Cultural Psychology of Education to the future of education: (a) the training of the psychologist as a technician and a researcher at the BA and MA university levels; (b) the training of the teacher who works in schools and universities; and (c) the research on education. Undoubtedly, this does not depend on individual initiatives, but on an environment and institutional culture geared toward interdisciplinary actions, where different voices make themselves heard—and the psychologist puts themselves in the position of providing an additional voice.

(a) The formation of the psychologist: Research and interdisciplinarity.

Part of the debate surrounding Cultural Psychology of Education deals directly with the problem of the training of the psychologist and, more specifically, the training of the psychologist as a technician, as a researcher, and as a public intellectual.

Cultural Psychology of Education assumes that the training of the psychologist involves a broad and deep access to the history of psychological knowledge and ideas, philosophy, social sciences and theories of education. Not to be confused with a simply panoramic and generic training, the cultural psychologist of education must have access to undergraduate and graduate curricula that impart a deep knowledge of epistemology, cultural studies and knowledge of developmental, systemic and ecosystemic approaches in human sciences.

In addition to engaging in strictly theoretical training, the cultural educational psychologist must be a researcher who masters methodologies of Cultural Psychology and developmental sciences and who has a critical awareness of the main theoretical, epistemological, political and methodological issues in psychological sciences at large.

A crucial objective is to provide access to broad training, complete with technical and interdisciplinary rigour, that is capable of cultivating a critical and non-dogmatic intellectual attitude in the student. The approach should allow practical involvement with different educational agents, such as students, families and the surrounding community, as well as the multitude of agents associated with schools and universities.

It is important that the psychology curricula enable the psychologist to work with educational policies and institutions in dealing with concrete situations that arise in the daily lives of educational agents and with the challenges of historically social, racial and economically unequal societies. Likewise, research training (for the investigation of concrete problems using rigorous methodological procedures, as well as conceptual research) must occupy a prominent place in this model.

Finally, the cultural educational psychologist needs to be open to intercultural and international relations in order to have the skills to exchange ideas, experiences and insights with colleagues of different nationalities and ethnicities. This multiculturalism is crucial for the training of the intellectual, the cultural educational psychologist in general and education in particular.

(b) Cultural Psychology of Education in teacher education.

The theoretical formulations of Cultural Psychology of Education, if taught in a critical way, can contribute both to teacher education (undergraduate courses and pedagogy) and to the continuing education of teachers who work directly in schools. According to Del Prette and Del Prette:

The psychological knowledge available on the fundamentals of education and teaching processes, on human relations, and on constructive alternatives in the promotion of professional and para-professional resources, allied to the knowledge of pedagogical, cultural, and political issues that characterize the current challenges of Education, give the psychologist a particularly desirable qualification for effective performance in this area (2002, p. 23).

This means that in addition to the teachers’ training at the university level, it is fundamental that the cultural educational psychologist contributes to continuing education programmes for teachers. In fact, the theories of psychological development and learning are indispensable references for understanding the teaching–learning process and the dynamics of relationships within the school institution. Likewise, understanding family relationships, the constitution of support networks, and family responsiveness can contribute to more efficient pedagogical strategies. The wisdom of Cultural Psychology over the last 40 years, accumulated in its various thematic areas and developed by researchers from different nationalities, is an important source for the understanding of education.

Cultural Psychology of Education has a necessary moral commitment to the dignity, integrity and diversity of human life. The promotion and protection of the well-being of individuals and diverse human communities is a core aspect of both the specific theoretical issues and the professional practices. Cultural educational psychologists are deeply interested in how people become human through education (Dazzani, 2016). Their investigation aims, goals and achievements are directly associated with the human cause and are always oriented toward human rights and to the defence of life.

(c) Research in Cultural Psychology of Education.

Research should cease to have a purely academic character and instead start dialoguing more intensely with the everyday life at schools, universities and other educational settings, seeking to penetrate into the spaces where the concrete lives of students, teachers and other educational agents take place. As a researcher, the psychologist should contribute: (a) to advancing knowledge in the fields of institutional relations, subjectivity and learning; (b) to understanding young people in their developmental trajectories without reducing them to the status of “student as recipient”; (c) to advancing the explanation of the various types of interactions that constitute the educational process; and (d) to studying the various phenomena that are unique to educational institutions in all their complexity.

Research in Cultural Psychology of Education must break down the boundaries between researchers and the location where the research takes place. Likewise, cultural educational psychologists must be prepared for frank and challenging dialogue with researchers from other cultural backgrounds, nationalities and fields of knowledge. They must have the capability to work in multicultural environments and to effectively deal with ethno-epistemologies; be willing to acquire skills in developing innovative and original methods and research designs adapted to local contexts; and be attentive to the application of the cultural psychological perspective to educational, professional and counselling contexts.

This demands that undergraduate and graduate programmes assume significant responsibility in the training of new psychologists who will work in the educational environment. New models of action are being created—models that are more compatible with the type of social organisation in which the subjects of education are born and develop, whether they are teachers, students, parents or technicians. It is largely up to the research conducted in psychology graduate courses to contribute to the advancement of knowledge and practice in the area of Cultural Psychology of Education.

Finally, the cultural educational psychologist should be seen as an intellectual, an agent who brings together competencies, responsibilities and intellectual autonomy. Among these competencies, the psychologist is expected to possess the following: an ability for international scientific publication; an ability to establish and maintain international research and professional networks; competence in multimodal qualitative analysis; competence in innovative interventions in community empowerment and participatory research; competence in critically interpreting, managing and transforming interdisciplinary and multicultural work contexts; and competence in applying Cultural Psychology to education and counselling.

Cultural Educational Psychology is relevant for working in a multicultural environment and for approaching social issues from an unconventional perspective. Thus, it can be functional to work in social science research, education and social work, community development, and international cooperation.

## Core contents and topics in Cultural psychology of education

The Cultural Psychology approach to education reflects the interdisciplinary nature of Educational Psychology and informs the applications of Educational Psychology in a variety of cultural contexts.

Educational Psychology today seems to have taken a back seat to theoretical research in order to prioritise more empirical and applicative concerns. This trend is only partly justified by the challenges facing educational systems worldwide, such as the multiculturalisation of classrooms and the increasing pace of innovation. The discipline’s response has focused on less theorisation and on medicalisation of the object of study. By “medicalisation” we mean the focus on performance and pathologies of learning, the obsession with quantitative assessment and the comparative and cross-cultural aspects of educational processes as a way of introducing a benchmark approach to education. All these concerns may be perfectly legitimate, but the focus on educational outcomes and performance has led Educational Psychology—the discipline that should study the processes of learning and teaching that are the most typical developmental phenomena of psychology—to approach its object as if it were a non-developmental phenomenon. On the other hand, we seem to have forgotten that some characters in psychology, such as Vygotsky, Lewin, Piaget and Bruner, understood in their development of major theories about the human psyche that Educational Psychology is a privileged field of study. These scholars not only intuited the relevance of educational processes to the development of the person, but they also understood that the theories, methodologies and questions that were raised in the study of education could provide fundamental knowledge to psychology in general. We desperately need renewed theorising in Educational Psychology. Therefore, on the one hand, we have a Psychology of Education that is producing too few theories for understanding a stock of empirical data, while on the other hand, we have a theoretical psychology that is not fully engaged in providing far-reaching theorising in education.

Cultural Psychology of Education is aimed at providing both an overview of the current trends in the field, especially outside the Anglo-American context, and a constant introduction of innovative and cutting-edge theoretical concepts. The focus is on the liminal phenomena in education (e.g., relationships, transitions and negotiations occurring between different contexts, such as school, family, formal and informal education, and work) (Marsico, 2018; Gomes et al., 2018). The emphasis on developmental processes, contexts, sense-making, theorising and borders places this scientific programme outside the current horizon of Educational Psychology.

## Major themes: Betweenness in education from a Cultural psychology perspective

As human beings, we live in a multifaceted world. We constantly create, regulate and traverse to modulate our relationships with the environment and with each other (Marsico et al., 2015). Here the role of education—with its liminal and always-future-oriented nature—undoubtedly occupies a leading position because it constantly works beyond the borders. Education is the outer frontier of human development, but it is the only frontier that is never ultimately crossed. It is a frontier of indeterminacy; it is our unreachable horizon that moves with us. With each step we take toward a new, higher level of education, the horizon also moves.

The idea beyond this conceptualisation of the educational process is the notion of *being on the move* within culturally organised life contexts (school, family, church, etc.) in specific spatio-temporal coordinates. Striving for uncertainty is an unavoidable characteristic of the human socio-cultural locomotion, which implies crossing the borders between different social settings within the insurmountable limit of irreversible time.

The borders condition evidences the relevance of the “space in between” (Marsico, 2016), which is a very challenging issue in education. Think, for instance, of some “in-between” aspects of education such as the daily (or periodic) migrations from school to home, from school to another school (music or dancing school), or from school to gym or playground. What discourses and practices saturate this interstitial zone and/or cross from one side to the other? How much (and what kind) of human activities, educational processes and social dynamics are made possible thanks to this betweenness? The crossing-borders phenomenon, which entails migrating from one place to another while living in the liminal condition, is an educational process in itself, but it has had a hard time being recognised as a relevant part of the individual educational enterprise. Yet its nature should be better investigated. For instance, what happens between home and school? (Marsico, 2018). Is the space in-between like a two-way boulevard or a one-way path? Assuming that there is a round trip between home and school, what is the nature of this daily (or recurrent) migration? Who migrates? What does it migrate and what not? Answering these questions requires assuming a very uncomfortable position on the border of the social context, *looking both inside and outside while studying the border zone.*

## Education in-between the established and the possible

One of the crucial, and still unsolved, issues in education is that of the universalities and particularities of the educational enterprise worldwide.

In the *Manifesto for the Future of Education* (2017), Marsico discussed the need for contemporary Cultural Psychology of Education to be increasingly international and global while promoting the cultural sensitivity of the educational intervention. In any society, the application of the know-how in the area of education is local. Cultural Psychology of Education intends to contribute to the discussion about education on a global scale (Szulevicz et al., 2016; Märtsin & Hviid, 2019).

The need to share and promote the relevant psychosocial processes, value systems, practices and ideologies in contexts that present a polyphony of perspectives is the very core of the contemporary debate in Psychology of Education from a cultural perspective.

We have recently become increasingly concerned with the question of what “diversity” or “inclusion” means in the context of the global migration flow that is changing the geography of the world, as well as the discussion of how current educational ideology can be disrespectful of alternative value systems and contribute to the impoverishment of cultural diversity around the world (Marsico, 2018). It is sufficient to think about the perpetration of acts of dominance, oppression and silencing of native cultures (Guimarães, 2017,).

Cultural Psychology of Education delves into the dilemma, addressed by Bruner (2007), of whether education (and schooling) should serve the established field of culturally acceptable knowledge or create the conditions for young generations to deal with the always-changeable future life. According to Bruner:

*I’ve become increasingly convinced that the powers of mind reach their fullness not simply in accumulationin what we come to knowbut rather in what we can do with what we know, how we are enabled to frame possibilities beyond the conventions of the present, to forge possible worlds* (2007, p. 2).

Cultural Psychology of Education has at its methodological and conceptual core not only the classic theme of becoming an individual and a member of society through education, but also the question of how to do it in a way that respects and supports the unique local culture while also paying attention to global trends. This leads directly to the issue of how to relate to the management of education with respect to values and alterity in a multicultural society. We urgently need an innovative look at the value–developmental processes that account for the complexity of ontogenesis in highly culturally-structured social settings such as school systems (Branco & Oliveira, 2018, ). Values at school cannot be produced by authority but only by dialogue. Values in education are the products of collective activity rather than emanations from abstract universals.

## Education and values

While scholars in the field of Developmental and Educational Psychology generally agree upon the relevance of the values in the ontogenesis of the human species, this topic is often disregarded in the current academic debate or—if considered—has been treated from a cross-cultural perspective that overlooks the endogenous process of value education and the local specificities. The cross-cultural value approach, indeed, focuses on the inter-individual differences between contexts using culture as an independent variable. Here, values are considered as given entities or fixed categories that can be studied or, even worse, “extracted” from individuals (Branco & Valsiner, 2012). Cultural Psychology of Education assumes the process of emergence and dialogical construction of values and alterity *in interaction*. Why did the values issue go through such a scientific underestimation? Probably because it calls for a definition of the horizon of human development and urges an answer to the question of “What are we educating for?” The perpetual educational dilemma reflects the tension between guiding and following human development.

This becomes incredibly important if we consider the global impact of the outcome hierarchies created by PISA Programme for International Student Assessment that have pushed many school systems into being tightly focused and regulated. The impact of testing systems has resulted in teaching becoming more focused on externally determined, specific success criteria. The foundations for teaching and learning have become distorted and driven by a deterministic output-regulated system, which is a detriment to the full development of children’s lives and learning.

Any discourse about values in the human development and educational practices evokes, then, the *phantasm* of who are the “men” of the future; what kind of human beings are we promoting? Also, the value issue dramatically shows the inadequacy of the scientific reductionism that permeates the contemporary academic world, almost incapable of a holistic perspective on human beings and their psychological functioning (Valsiner et al., 2016, Tateo & Marsico, 2018) which, instead, is the very core of the Cultural Psychology perspective.

## Teaching, learning, and assessment in Cultural psychology of education

The learning goals of the Cultural Psychology of Education curriculum at the BA and MA levels include solid bases in the epistemology of Developmental Psychology as well an extended humanities and social sciences background. By the end of the BA and MA programmes in Psychology, the student should have acquired an extensive knowledge of the main approaches in the fields of Cultural Psychology and developmental sciences’ main theories. The student should have also developed skills to operate in multicultural settings and to manage cultural diversity.

At the doctoral level of the programme in Cultural Psychology of Education, the student should acquire advanced knowledge of the Cultural Psychology of Education theory, epistemology and methodology, as well as a robust interdisciplinary knowledge of the developmental sciences and of the study of human activity. The doctoral student should also be competent in critically interpreting, managing and transforming interdisciplinary and multicultural contexts and in being capable of networking at the international level.

For a majority of students, the traditional lecture has limitations in provoking high levels of understanding and performance. Yet there are alternatives for reaching these outcomes even in the large lecture-based teaching system, such as implementing interactive forms of lecturing that comprise work with concept maps, forming learning partnerships with other students, writing minute papers and the strategic changing of activities within lectures. Another aspect of teaching is particularly necessary in promoting a functional understanding that links knowledge to concrete situations in professional contexts. Relevant teaching and learning activities involve students in case-based and problem-based pedagogical scenarios to apply knowledge to given cultural domains.

Teaching and learning in the workplace, such as through internships (Mele et al., 2021), are of particular importance for Cultural Psychology of Education since they provide opportunities to apply newly acquired knowledge to real educational practice.

## Generalizing conclusion

The following are fundamental principles that can be assumed as guidelines to make the teaching Educational Psychology truly Cultural.

1. To examine the topic of globalisation and standardisation of education through a renewal cultural developmental and educational perspective. Case studies and examples from varied cultural contexts might be a good starting point, but they should serve a theoretical advancement in the field.
2. To rethink the relationship between actors, practices and borders within different educational contexts. Cultural contexts of various intercontinental educational systems might be considered to this scope, but only for the sake of getting a generalizable understanding of the “in-between” educational processes.
3. To revise the process of identity construction in the school setting and all its cultural components. This implies a critical revision of what are commonly called dysfunctional processes (i.e. disruptive behaviours, drop-out etc.) in school contexts.
4. To design and adopt new methodological approach for researching Cultural Psychology of Education that are sensitive to the social ecology of the local filed under investigation.

We have tried to draft the above mentioned principles in the form of possible actions to take when we want to design, develop, and implement curricula that ensure the equity, quality, development-relevance and resource efficiency of education and learning systems.

## Data Availability

Data sharing not applicable to this article as no datasets were generated or analysed during the current study.

## References

[CR1] Branco AU, Lopes-de-Oliveira MC (2018). Human Development Within Educational Contexts: Alterity, Values and Socialization.

[CR2] Branco AU, Valsiner J, Branco AU, Valsiner J (2012). Values as culture in self and society. Cultural psychology of values.

[CR3] Bruner, J. S. (2007). *Cultivating the possible, address at Oxford dedication*. Jerome Bruner Building

[CR4] http://www.education.ox.ac.uk/wordpress/wp-content/uploads/2011/03/Transcript-Cultivating-the-

[CR5] Possible.pdf

[CR6] Dazzani, V. (2016). Education: The process of becoming. In J. Valsiner, G. Marsico, N. Chaudhary, T. Sato, & V. Dazzani (Eds.), *Psychology as the science of human being: The Yokohama Manifesto, Annals of theoretical psychology, 13* (pp. 337–348). Springer

[CR7] Del Prette, Z. A. P., & Del Prette, A. (2002). Psicología de las habilidades sociales: terapia y educación.Revista Evaluar, *3*(1)

[CR8] Gomes R, Dazzani V, Marsico G (2018). The role of responsiveness within the self in transitions to university. Culture & Psychology.

[CR9] Guimarães, D. S. (2017). Amerindian psychology: Cultural basis for general knowledge construction. In B.Wagoner, I. Bresco, & S. H. Awad (Eds.), The psychology of imagination: History, theory and new

[CR10] research horizons (pp.221–238). Charlotte:Information Age Publishing

[CR11] Hviid, P., & Märtsin, M. (Eds.). (2019). (Eds). *Culture in education and education in culture: Tensioned dialogues and creative constructions, Cultural Psychology of Education* (10.). Springer

[CR12] Mele, E., Español, A., Carvalho, B., & Marsico, G. (2021). Beyond technical learning: internship as a liminal zone on the way to become a psychologist. *Learning, Cultural and Social Interaction;*10.1016/j.lcsi.2020.100487

[CR13] Marsico G (2016). The Borderland. Culture & Psychology.

[CR14] Marsico G (2018). The Challenges of the Schooling from Cultural Psychology of Education. Integrative Psychological and Behavioural Sciences.

[CR15] Marsico, G., Dazzani, V., Ristum, M., & Bastos, A. C. (Eds.). (2015). *Educational contexts and borders through a cultural lens – Looking inside, viewing outside, Cultural Psychology of Education* (1 vol.). Springer

[CR16] Mele E, Español A, Carvalho B, Marsico G (2021). Beyond technical learning: Internship as a liminal zone on the way to become a psychologist. Learning Culture and Social Interaction.

[CR17] Szulevicz, T., May Eckerdal, R., Marsico, G., & Valsiner, J. (2016). When disruptive behaviour meets outcome-based education. *Psihologij*a. Vol. 49(4),447–468, DOI: 10.2298/PSI1604447S

[CR18] Tateo L, Marsico G (2018). The synthetic and syncretic nature of human culture. Human Arenas.

[CR19] Valsiner, J., Marsico, G., Chaudhary, N., Sato, T., & Dazzani, V. (Eds.). (2016). Psychology as the science of human being: The Yokohama Manifesto*Annals of Theoretical Psychology*. Springer

